# Physiological Occlusal Force Promotes Heme Oxygenase-1 to Repair Periodontal Injury in a Rat Model of Replanted Teeth

**DOI:** 10.1016/j.identj.2026.111101

**Published:** 2026-09-03

**Authors:** Weiqi Jia, Xiaohan Liu, Chang Wu, Chuanyezi Wu, Liming Jiang

**Affiliations:** a Department of Pediatric Dentistry, School and Hospital of Stomatology, China Medical University, Shenyang, Liaoning, China; b Liaoning Provincial Key Laboratory of Oral Diseases, Shenyang, Liaoning, China,; c Department of Prosthodontics, School of Stomatology, China Medical University, Shenyang, Liaoning, China

**Keywords:** Occlusal force, Replanted tooth, Heme oxygenase-1

## Abstract

**Objective:**

The physiological occlusal force generated by mastication plays a critical role in tooth replantation after complete avulsion by promoting periodontal ligament healing. Heme oxygenase-1 (HO-1), a classical cytoprotective factor, exhibits mechanoresponsive expression in periodontal ligament cells (PDLCs). This study aims to investigate whether occlusal force can reduce replacement root resorption during the healing of periodontal ligament in replanted teeth and its potential mechanisms.

**Methods:**

A tooth avulsion model was established in occlusal group rats and occlusal deprivation group rats. The healing of periodontal ligament was evaluated at 7 and 28 days post-replantation using micro-computed tomography, histological analysis, and immunohistochemistry. PDLCs were isolated to construct the injured periodontal ligament cells (iPDLCs) model. Following the application of cyclic tensile force, collagen synthesis level and the expression changes of HO-1 and transforming growth factor-beta 1 (TGF-β1) were assessed.

**Results:**

The physiological occlusal force reduced replacement root resorption, promoted periodontal healing and the conversion of type III collagen (COL Ⅲ) to type I collagen (COL Ⅰ), and increased HO-1 expression in the periodontal ligament of replanted rat teeth. The cyclic tensile force (4% strain, 0.5 Hz) applied for 4 hours upregulated the expression of HO-1, COL Ⅰ, and COL III in iPDLCs. Enhanced HO-1 expression increased the levels of TGF-β1, COL I, and COL III, whereas inhibition of HO-1 or blockade of TGF-β1 reduced COL I and COL III expression. Simultaneous upregulation of HO-1 and inhibition of TGF-β1 resulted in decreased COL I and COL III expression.

**Conclusion:**

Physiological occlusal force may be associated with reduced replacement root resorption and elevated HO-1 expression, while also promoting collagen synthesis in the injured periodontal ligament. Low deformation with short-term cyclic tensile force induces HO-1 expression, thereby promoting COL I and COL III synthesis in iPDLCs. This process is closely associated with the TGF-β1 signaling pathway.

## Introduction

Tooth avulsion is a common type of dental trauma and the second leading cause of tooth loss in children.^1^^,^^2^ Currently, tooth replantation is the primary treatment for avulsed permanent teeth.^3^ The prognosis of replantation is closely associated with the viability of periodontal ligament cells (PDLCs), because PDLCs damage under extraoral conditions often leads to poor outcomes and compromised tooth retention.^3^ Consequently, strategies for repairing injured PDLCs and promoting periodontal ligament (PDL) healing remain key focuses of clinical research.

Occlusal force plays a vital role in maintaining the normal structure and function of the PDL. Occlusal deprivation induces PDL atrophy, characterized by reduced cell numbers, disorganized collagen fiber arrangement, and diminished vascularization.^4^ The current consensus is that occlusal loading is critical for PDL healing in replanted teeth. Compared to rigid fixation, elastic fixation better mitigates inflammatory root resorption and osteoclast formation, thereby enhancing the likelihood of PDL healing.^5^^,^^6^ The International Guidelines for Dental Trauma (2020) also recommend the use of flexible splints to stabilize replanted teeth while permitting slight mobility, which facilitates PDL healing.^3^^,^^7^ Nevertheless, the mechanistic basis by which a physiological occlusal force regulates injured PDLC repair and promotes PDL healing remains poorly understood.

Heme oxygenase-1 (HO-1) is a well-established cytoprotective enzyme that catalyzes the oxidative cleavage of heme at the α-methene bridge, yielding carbon monoxide, biliverdin, and free ferrous iron, thereby mediating its cytoprotective and antioxidant functions.^8^, ^9^, ^10^ In PDLCs, HO-1 is a critical regulator of cell survival and apoptosis inhibition. Under inflammatory and oxidative stress conditions induced by lipopolysaccharide stimulation, HO-1 exerts anti-apoptotic effects by downregulating pro-apoptotic BCL2-associated X protein (Bax) and concurrently upregulating anti-apoptotic B-cell lymphoma-2 (Bcl-2) expression.^11^^,^^12^ Furthermore, HO-1 exhibits potent anti-inflammatory activity by suppressing pro-inflammatory cytokine production.^13^ Following tooth avulsion, PDLCs undergo progressive apoptosis due to the combined insults of desiccation and nutrient deprivation, leading to a significant impairment of cellular viability.^14^^,^^15^ Notably, storage media for avulsed teeth and root surface treatment agents can attenuate oxidative stress damage by upregulating HO-1 expression and reducing the generation of reactive oxygen species, thereby inhibiting root resorption in replanted teeth.^16^^,^^17^ *In vitro* studies have demonstrated that cyclic tensile strain significantly upregulates HO-1 expression in PDL stem cells.^18^ Our previous proteomic analysis confirmed that mechanical loading enhances HO-1 expression in PDL cells. However, the specific role of the physiological occlusal force in promoting PDL healing through HO-1-mediated repair mechanisms remains to be elucidated.

Accumulating evidence demonstrates the pivotal role of collagen in periodontal tissue repair. Following PDLCs injury, the osteogenic differentiation capacity of PDLCs increases, whereas their fibroblastic differentiation potential decreases.^19^ During PDL tissue healing, mechanically stimulated injured PDLCs primarily contribute to collagen synthesis and secretion. Experiments simulating various storage media for replanted teeth revealed the decreased expression of type I collagen (COL Ⅰ) in PDLCs.^20^ Transforming growth factor-β1 (TGF-β1), a multifunctional cytokine abundantly expressed in PDL tissue, participates in diverse cellular processes including differentiation, proliferation, migration, and extracellular matrix (ECM) synthesis. Elevated TGF-β1 levels effectively promote periodontal tissue regeneration.^21^ In PDLCs, TGF-β1 enhances collagen production by upregulating Smad2/3 phosphorylation and improves cell migratory capacity.^22^ TGF-β1 also induces fibroblastic differentiation in PDL stem cells while regulating the synthesis and secretion of ECM components, including collagen.^23^ Notably, intermittent compressive stress increases TGF-β1 expression in PDL cells, suggesting its involvement in mechanotransduction-mediated cellular responses.^24^

Therefore, this study aims to establish a rat model of tooth avulsion and replantation to elucidate the role of physiological occlusal force generated by mastication in PDL repair and to investigate *in vitro* whether cyclic tensile force promotes type I and type III collagen (COL III) synthesis in iPDLCs through a mechanism involving HO-1 and the TGF-β1 signaling pathway. These findings provide a theoretical foundation for understanding the mechanisms contributing to the functional recovery of the injured PDL and for identifying therapeutic targets in tooth replantation.

## Materials and methods

### Establishment of the rat model of tooth replantation

This study was approved by the Institutional Animal Care and Use Committee (IACUC) of China Medical University (approval No.: CMU20240623). The sample size for animal experiments was calculated based on the literature, with eight rats per group.^25^ A total of 32 four-week-old Sprague-Dawley male rats weighing 110 ± 10 g were randomly assigned to either the occlusion group or the occlusal deprivation group. The rats were housed in a specific pathogen-free environment with a constant temperature of 25°C and a 12-hour light/dark cycle. They had free access to water and food, with bedding, water, and feed replenished every 2 to 3 d. After one week of acclimatization, the rats were weighed and anesthetized via an intraperitoneal injection of 1% sodium pentobarbital solution. The left maxillary first molar was used as the experimental tooth. Following extraction, the tooth was immediately placed in physiological saline and replanted to its original position after 5 min. The right maxillary first molar of each rat served as the control (no intervention). In the occlusion group, no further treatments were administered after replantation. In the occlusal deprivation group, the antagonist tooth was extracted after replantation. The rats were fed a soft diet for 3 days postoperatively, and each group received an intramuscular injection of penicillin sodium (20,000 IU) once daily for 3 consecutive days.^26^

### Micro-CT analysis

**Fig. 1:**
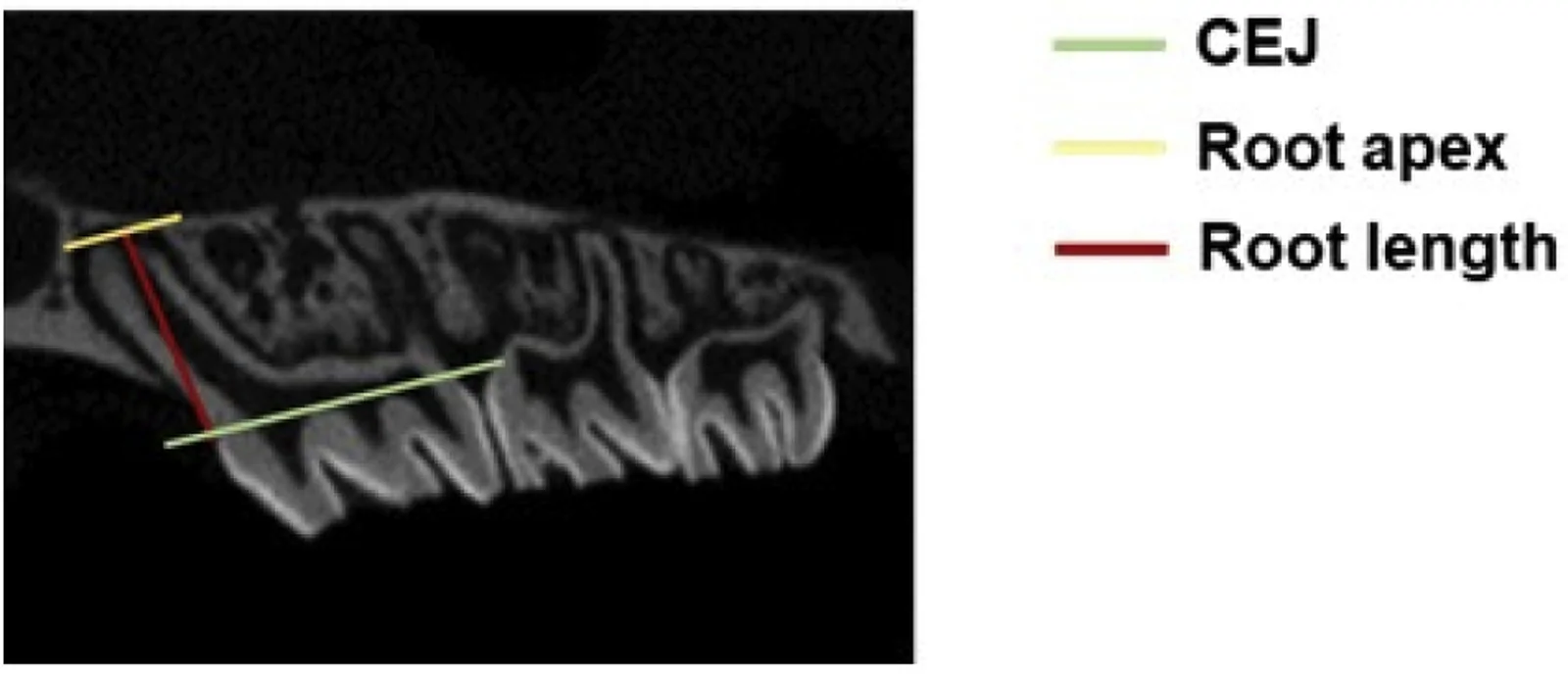
Schematic diagram of the length measurement of the mesial root of the maxillary first molar in Micro-CT images. CEJ: cementoenamel junction.

A blinded examiner with good reliability performed the micro-CT acquisition (SkyScan 1276, Belgium) and analysis. Maxillary bone samples were scanned using a micro-CT system with the following parameters: voltage 85 kV, current 200 μA, and exposure time 386 ms. After scanning, the DataViewer software was used to capture cross-sectional images at the mid-root level (1/3) of the mesial root of the maxillary first molar. The widest PDL width was measured using the ImageJ software. Sagittal images showing the maximum length of the mesial root were analyzed. Linear measurements were taken from the cementoenamel junction to the apex of the mesial root (mesial root length, mRL; Figure 1).^26^^,^^27^

The mRLs of the left (replanted) and right (control) first molars were assumed to be equal for each rat. The percentage reduction in root length was calculated as follows:

$$\text{Root}\;\text{length}\;\text{reduction}\;=\;\frac{\text{RightcontrolmRL}\;-\;\text{LefttoothmRL}}{\text{RightcontrolmRL}}\;\times \;100\%$$

### Histological staining

The rats were euthanized 7 or 28 days postoperatively (*n* = 8). The tissues were dissected, and excess muscle and oral mucosa were removed while preserving the maxilla. Samples were fixed in 4% paraformaldehyde (Servicebio, China) and decalcified in 10% EDTA (Boster, China), with the decalcification solution replaced every 2 to 3 days. Decalcification was completed after approximately 8 weeks, followed by paraffin embedding and sectioning at 3.5 μm thickness. The sections were dehydrated using an ethanol gradient and stained with H&E, picrosirius red, and immunofluorescence. For immunofluorescence, the sections were treated with a 1:200 dilution of anti-HO-1 primary antibody (Abcam, USA). Sirius red staining was examined under a polarized light microscope with identical polarization conditions, and images were captured. The areas of COL I (orange-yellow) and COL III (green) in the polarized light images were separated and extracted using Photoshop(version 19.0, Adobe Systems). It was assumed that the collagen fibers of the periodontal ligament consist of type I and type III collagen. The proportions of COL I and COL III in the periodontal ligament were calculated using the following formula:

$$\text{Proportions}\;\text{of}\;\text{COL}\;I=\frac{\text{COL}\;I}{\text{COL}\;I\;+\;\text{COL}\;\text{III}}\times \;100\%$$

$$\text{Proportions}\;\text{of}\;\text{COL}\;\text{III}=\frac{\text{COL}\;\text{III}}{\text{COL}\;I+\text{COL}\;\text{III}}\times \;100\%$$

HO-1 immunofluorescence staining images were captured using an inverted fluorescence microscope. The number of positively stained cells for each fluorescence marker was counted using ImageJ (version 1.50i, NIH), and the percentage of HO-1-positive cells was calculated. During image acquisition and analysis, the investigators were completely blinded to the group assignments. All measurements were repeated three times, and the final values were expressed as the average.

### Cell isolation, culture, and identification

These experiments were approved by the Ethics Committee of China Medical University School of Stomatology (K2023054). Human PDL tissues from the middle root third were collected, washed three times with phosphate-buffered saline, and digested with type I collagenase and dispase at 37°C for 30 min. The cell suspension was seeded in six-well plates and cultured in α-MEM (Servicebio) supplemented with 20% fetal bovine serum and 100 U/mL penicillin-streptomycin (Servicebio). Cells migrated from the explants after 7 days. Upon reaching 80% confluence, cells were passaged and maintained in α-MEM with 10% fetal bovine serum and 100 U/mL penicillin-streptomycin at 37°C and 5% CO_2_. Cells from passages 3 to 6 were used in the experiments.

For immunocytochemistry, PDLCs from passage 3 were seeded on coverslips (14 mm diameter) in six-well plates. At 60% to 70% confluence, the cells were fixed with 4% paraformaldehyde, blocked with goat serum, and incubated overnight with anti-vimentin (1:500) or anti-cytokeratin (1:500) primary antibodies (Proteintech, China). After incubation with horseradish peroxidase-conjugated secondary antibodies (1:1000, Proteintech) for 1 h at room temperature, staining was visualized using DAB substrate (Genenode, China). The sections were counterstained with hematoxylin for 3 min and mounted with a neutral resin.

### Establishment of iPDLCs, trypan blue staining, and flow cytometry

Extracted teeth were air-dried for 0, 15, 30, or 60 min, followed by digestion with type I collagenase and dispase at 37°C for 30 min. The cell suspension was centrifuged at 1200 rpm for 5 min to isolate PDLCs. To simulate dry storage conditions, cells were exposed to sterile air for 0, 5, 10, or 15 min before trypsinization. Cell viability was assessed by trypan blue staining (Beyotime, China). For apoptosis analysis, cells were resuspended in binding buffer and stained with 10 μL annexin-V and 10 μL propidium iodide for 10 min in the dark. The number of apoptotic cells was quantified using a flow cytometer (FACSCanto II; BD Biosciences, USA), and data were analyzed using the FlowJo software.

### Cyclic tensile force application

PDLCs (passages 3-8) were seeded at a density of 2 × 10^5^ cells/well in BioFlex six-well plates and divided into iPDLC strain, iPDLC control (no strain), and PDLC groups. The Flexcell 5000T system was programmed with the following parameters: channel 2; strain type: cyclic tensile; waveform: sinusoidal; frequency: 0.5 Hz; elongation: 4%, 6%, or 10%; duration: 2 or 4 h. Non-strained control cells were cultured under identical conditions at 37°C and 5% CO_2_.

### Protein extraction and western blotting

Total protein was extracted using radioimmunoprecipitation assay (RIPA) lysis buffer containing PMSF. Primary antibodies against HO-1, COL I, COL III, and GAPDH were used. Protein bands were visualized using an ECL HRP substrate kit.

Cells were lysed in RIPA buffer (Beyotime) containing 1% PMSF and sonicated on ice. Lysates were centrifuged at 12,000 × *g* for 15 min, and protein concentrations were determined using a BCA kit (Beyotime). Samples were mixed with 5× loading buffer, denatured at 95°C for 15 min, and separated using SDS-PAGE. After transfer, membranes were blocked with 5% bovine serum albumin and incubated overnight with primary antibodies against HO-1 (1:1000), COL I (1:1000), COL III (1:1000), TGF-β1 (1:1000), and GAPDH (1:4000). Horseradish peroxidase-conjugated secondary antibodies (1:1000, Proteintech) were applied, and chemiluminescence was detected using a Tanon 5200 system (China). Band intensities were quantified using the ImageJ software. The intensity of each target protein band was normalized to the intensity of the corresponding GAPDH band from the same lane and same membrane.

### Statistical analysis

Data are presented as the mean ± standard deviation. Statistical analyses were performed using SPSS 23.0. Independent *t*-tests were used for two-group comparisons. If the samples between groups are from the same experimental animals, the paired *t*-test is used. Shapiro-Wilk test was used to assess normality. Normally distributed data were analyzed by independent *t*-test (two groups) or paired *t*-test (differences normal). Otherwise, Mann-Whitney U (independent) or Wilcoxon signed-rank (paired) tests were applied. All tests were two-tailed with significance at *P* < .05.

## Results

### Physiological occlusal force reduces replacement root resorption in a rat model of replanted teeth

To observe the effects of physiological occlusal force generated by mastication on root resorption and PDL healing in replanted teeth, we established a tooth replantation model in Sprague–Dawley rats using the left maxillary first molar. Micro-CT was performed on postoperative day 28.

**Fig. 2:**
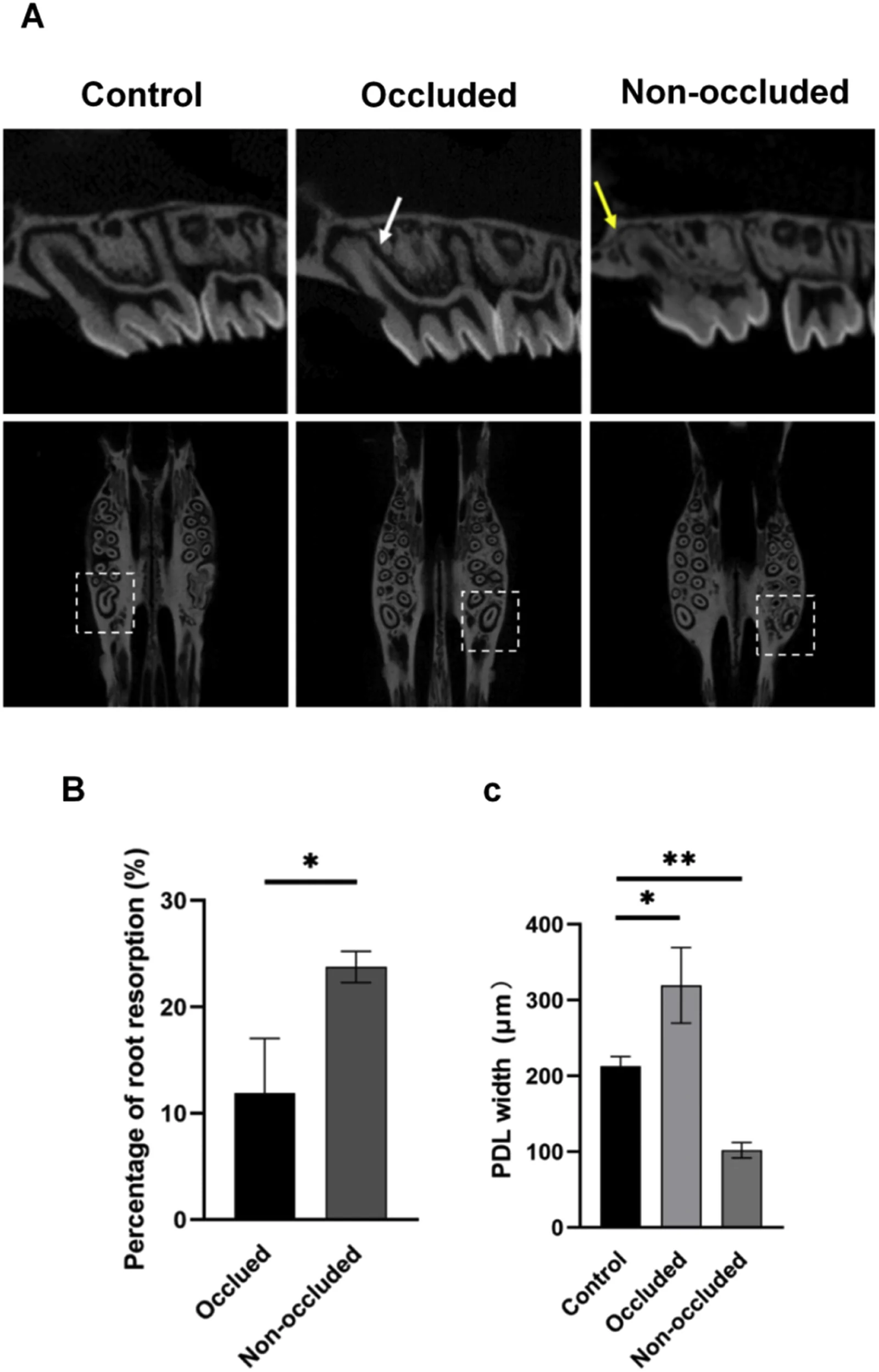
Root resorption of replanted teeth under reduced physiological occlusal force. **A** Micro-CT images of the sagittal and horizontal sections of the first molar 28 days after tooth replantation. In the control group, the periodontal ligament space appears as a low-density translucent area, intact and clear, with no signs of root resorption. The occlusion group exhibits slight resorption at the apical third of the root surface (white arrow) and a slightly increase in periodontal ligament width. The occlusal deprivation group shows replacement root resorption (yellow arrow), with partial root replacement by alveolar bone and a marked reduction in periodontal ligament width. **B** Percentage reduction in root length of the mesial root of the first molar on day 28 after replantation (*n* = 3). **C** Periodontal ligament width of the mesial root of the first molar on day 28 after replantation (*n* = 3). *P < .05, **P < .01*.* Error bars represent the mean ± standard deviation.

Imaging results showed that, in the occlusion group, only minimal root resorption was observed in the apical third of the mesiobuccal root, with a clear and intact PDL space slightly wider than that in the control group. In contrast, the occlusal deprivation group exhibited a narrowed PDL space with partial loss of ligament continuity and radiographic evidence of tooth-to-bone contact in the apical region, indicating replacement root resorption (Figure 2A).

Quantification of root resorption revealed that the occlusion group had 9.52 ± 3.66% root resorption, whereas the occlusal deprivation group showed 23.78 ± 1.46% root resorption. This significant difference suggests that physiological occlusal force can reduce replacement root resorption in replanted teeth (Figure 2B).

Measurements of the PDL width in the middle third of the mesiobuccal root demonstrated that the control group had an average width of 213.00 ± 12.77 μm. The occlusion and occlusal deprivation groups showed PDL widths of 319.67 ± 49.81 μm and 102.00 ± 10.15 μm, respectively. Compared to the control group, the occlusal deprivation group showed narrowing of the PDL space. The width of PDL space in occlusion group increased slightly, but there was no statistical difference. (Figure 2C).

### Physiological occlusal force promotes periodontal tissue repair in the rat model of replanted teeth

**Fig. 3:**
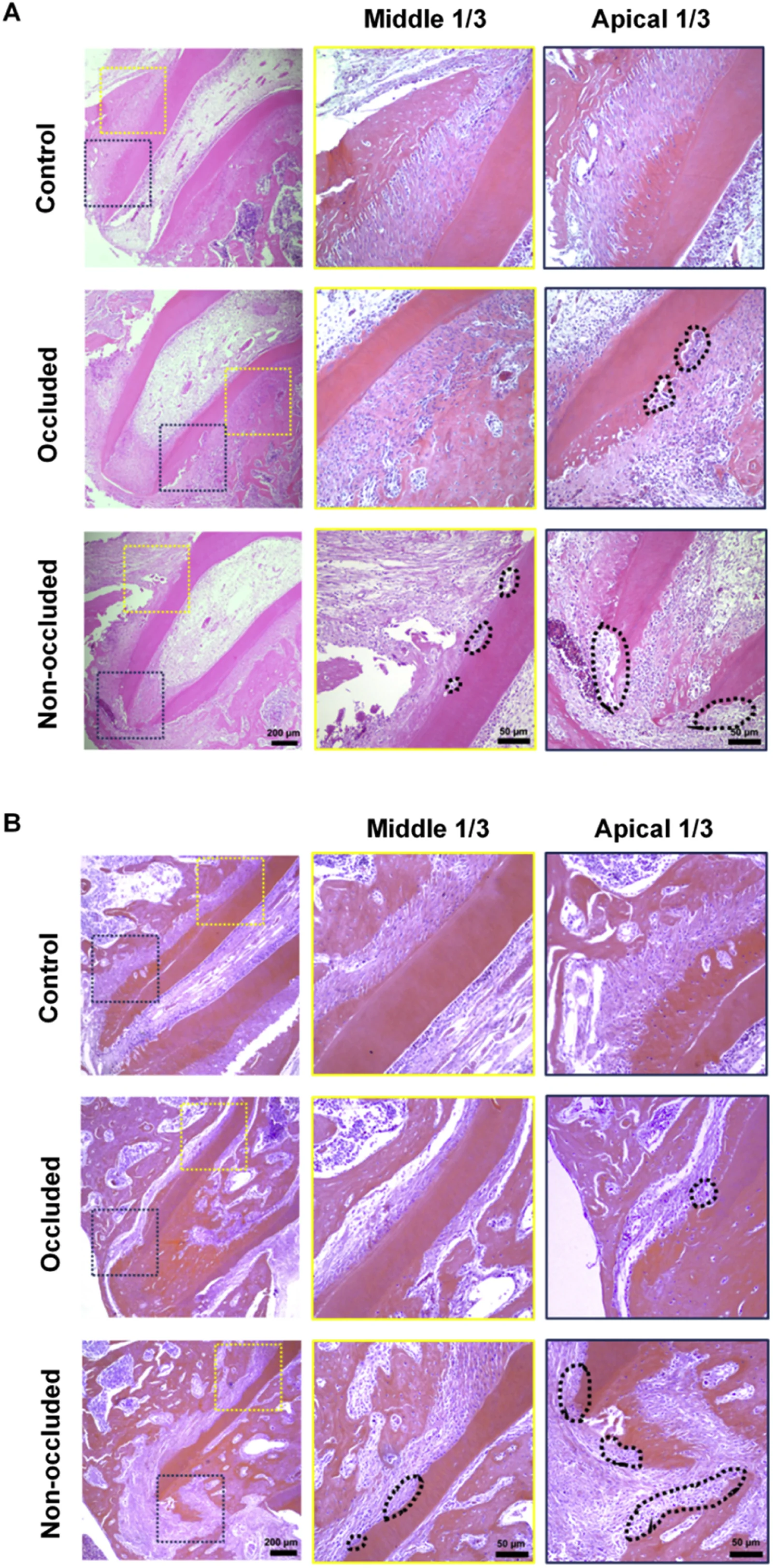
Physiological occlusal force promotes periodontal ligament healing and reduces root resorption in replanted teeth. **A** Hematoxylin and eosin (H&E) staining 7 days after replantation reveals irregularly arranged periodontal ligament fibers and scattered resorption lacunae at the apex in the occlusion group compared to the control group. The occlusion deprivation group exhibits disorganized periodontal ligament fibers and resorption lacunae at the middle and apical root regions (black circles) (*n* = 8). **B** H&E staining 28 days after replantation shows regularly arranged periodontal ligament fibers in the occlusion group. The occlusion deprivation group displays root resorption at the middle region, communication between pulp and periodontal tissues, and disorganized periodontal ligament fibers with extensive root resorption at the apex (black circles) (*n* = 8).

To further observe changes in the periodontal tissues of the replanted teeth, we performed hematoxylin and eosin (H&E) staining on samples from rats of the three study groups at different postoperative time points. The staining results at 7 days postoperatively showed that in the control group, the PDL fibers of the mesiobuccal root of the first molar were regularly and orderly arranged with intact root surfaces and no resorption. Compared to the control group, the occlusion group exhibited a slightly irregular arrangement of PDL fibers in the middle root region, intact root surfaces, and a small amount of inflammatory cell infiltration. In the apical region, the PDL fibers showed less regularity than those in the middle root region, with the presence of small resorption lacunae and inflammatory cell infiltration. In contrast to the occlusion group, the occlusal deprivation group showed discontinuous fiber structures in the middle root region, a significantly disorganized fiber arrangement in the apical region, and visible resorption lacunae in both middle and apical regions, with more pronounced resorption lacunae and inflammatory cell infiltration in the apical region (Figure 3A).

Staining results at 28 days postoperatively revealed that, in the occlusion group, regularly arranged PDL fibers were observed in both the middle and apical regions of the mesiobuccal root of the first molar, with no significant inflammatory cell infiltration and only scattered small resorption lacunae visible in the apical region. The occlusal deprivation group exhibited significant root resorption with unclear boundaries between pulp and PDL tissues in some areas of the middle root region, bone-like structures distributed within the periodontal ligament, and relatively regularly arranged PDL fibers visible in the apical region (Figure 3B).

### Physiological occlusal force modulates the ratio of type I to type III collagen in the PDLs of replanted teeth in the rat model

**Fig. 4:**
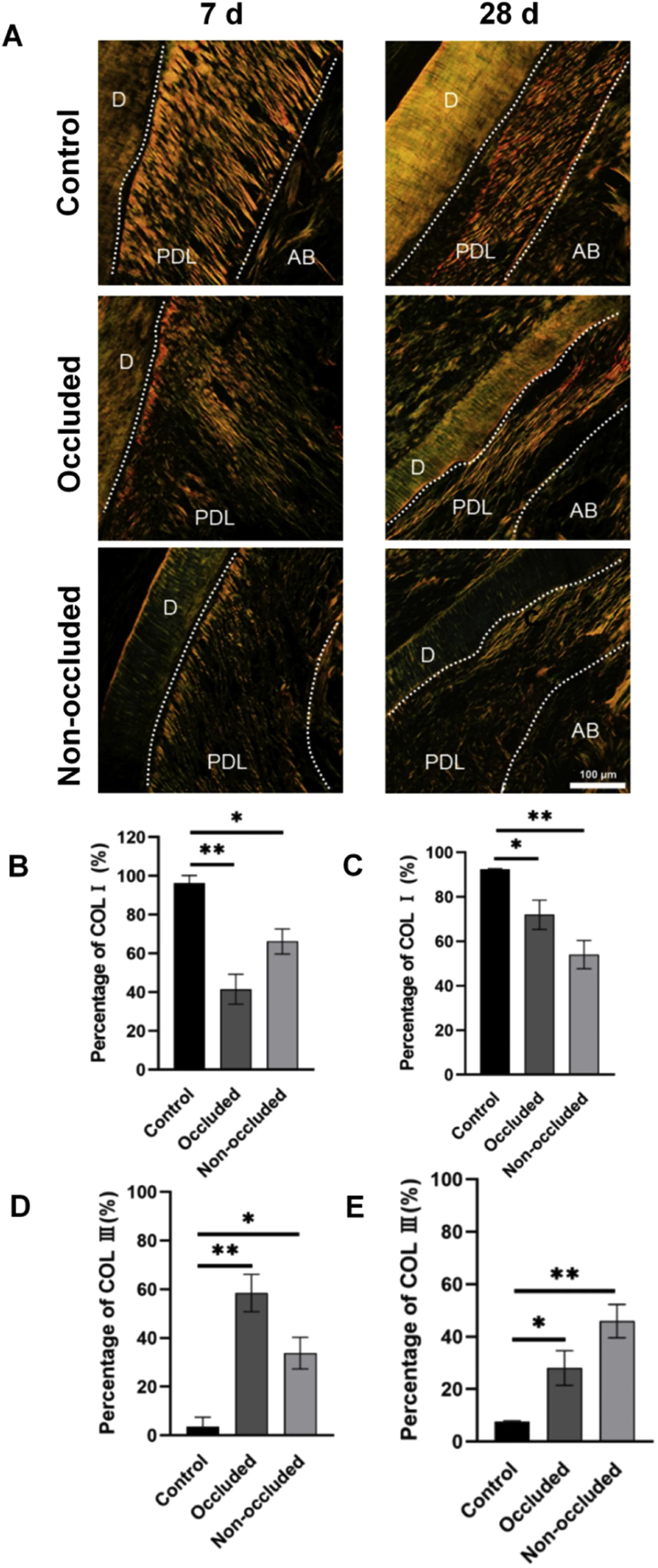
Physiological occlusal force modulates the ratio of COL I to COL III expression in the periodontal ligaments of replanted teeth. **A** Picrosirius red staining 7 and 28 days after replantation shows a significant decrease in COL I expression (orange-yellow) and an increase in COL III expression (green) in both the occlusion and occlusion deprivation groups compared to the control group. The occlusion group exhibits a higher proportion of COL III expression than the occlusion deprivation group. On day 28, COL I expression remains significantly reduced, whereas COL III levels remain elevated in both groups, but the occlusion group shows a ratio closer to that of the control group. **B, D** Ratio of COL I and COL III levels in the mesial root of the first molar on day 7 after replantation (*n* = 3). **C, E** Ratio of COL I and COL III levels in the mesial root of the first molar on day 28 after replantation (*n* = 3). AB: alveolar bone; D: dentin; PDL: periodontal ligament. Scale bar = 100 μm; *P < .05, **P < .01*.* Error bars represent the mean ± standard deviation.

To investigate the specific aspects of periodontal tissue healing influenced by the physiological occlusal force during the healing process, we performed Sirius red staining to observe changes in the expression of COL I and COL III collagen in the periodontal tissues of the replanted teeth (Figure 4A) and quantitatively compared the collagen ratios.

The staining results at 7 days postoperatively showed that in both the control and occlusal deprivation groups, the content of COL I decreased, whereas that of COL III increased in the PDL tissues of the mesiobuccal root of the first molar. According to the semi-quantitative analysis of collagen ratios, the control group had a COL I ratio of 96.40 ± 3.89% and a COL III ratio of 3.61 ± 3.89%, the occlusion group had a COL I ratio of 41.52 ± 7.68% and a COL III ratio of 58.48 ± 7.68%, and the occlusal deprivation group had a COL I ratio of 66.20 ± 6.50% and a COL III ratio of 33.80 ± 6.50%.

Compared to the control group, both the occlusion and occlusal deprivation groups showed significant decreases in COL I expression and increases in COL III expression. However, compared to the occlusal deprivation group, the occlusion group exhibited a more pronounced increase in COL III levels. These changes in collagen ratios suggest that physiological occlusal force is associated with increased synthesis of COL III during PDL healing in replanted teeth (Figure 4B, D).

On postoperative day 28, the semi-quantitative analysis showed that the control group had a COL I ratio of 92.39 ± 0.35% and a COL III ratio of 7.61 ± 0.35%, the occlusion group had a COL I ratio of 71.99 ± 6.59% and a COL III ratio of 28.01 ± 6.59%, and the occlusal deprivation group had a COL I ratio of 54.02 ± 6.32% and a COL III ratio of 45.98 ± 6.32%. Compared with the control group, both the occlusion and occlusal deprivation groups demonstrated significantly decreased COL I ratios and significantly increased COL III ratios. Notably, the COL I levels in the occlusion group were similar to those in the control group. Therefore, combined with the results at 7 days postoperatively, these findings indicate that a physiological occlusal force promotes the expression of COL III and facilitates the conversion of COL III to COL I in the periodontal tissues of replanted teeth (Figure 4C, E).

### Physiological occlusal force promotes HO-1 expression in the PDLs of replanted teeth in rats

**Fig. 5:**
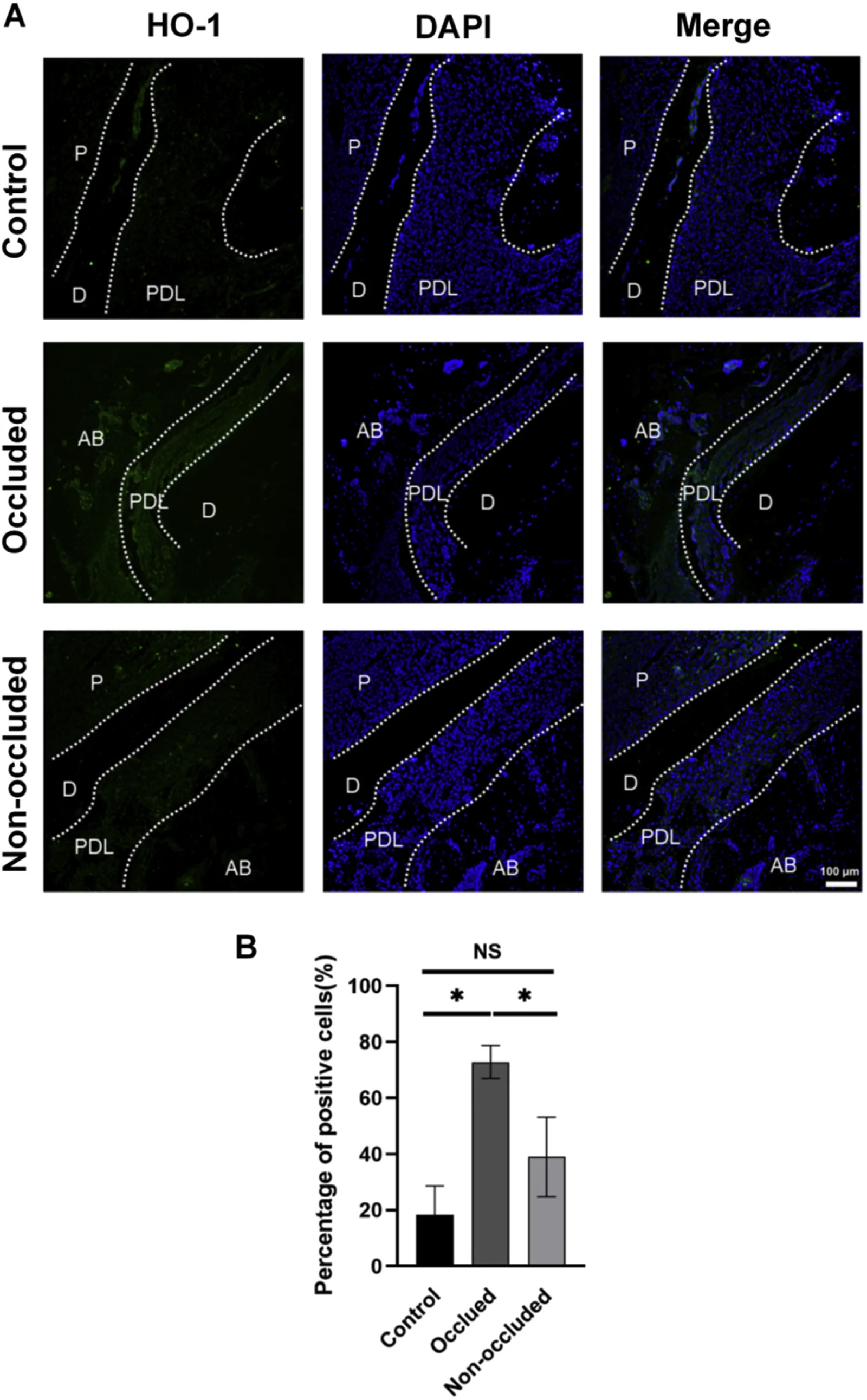
Physiological occlusal force increases HO-1 expression in the periodontal ligament of replanted teeth. **A** 7 days after replantation, HO-1 expression in the periodontal ligament of the mesial root is higher in the occlusion group than in the control and occlusal deprivation groups. **B** Count of HO-1 immunofluorescence-positive cells (*n* = 3). AB: alveolar bone; D: dentin; PDL: periodontal ligament; P: dental pulp. Scale bar = 100 μm; NS: *P* > .05, *P < .05. Error bars represent the mean ± standard deviation.

To investigate the potential functional proteins and their expression patterns during PDL healing of replanted teeth under physiological occlusal force, we performed immunofluorescence staining of the tissue samples. The immunofluorescence staining results on postoperative day 7 demonstrated the following HO-1-positive cell ratios: control group, 18.30 ± 10.36%; occlusion group, 72.81 ± 5.90%; and occlusal deprivation group, 38.97 ± 14.19%. The expression level of HO-1 in PDL tissues was significantly higher in the occlusion group than in the control and occlusal deprivation groups (Figure 5B). These findings suggest that physiological occlusal force enhances HO-1 expression in the PDLs of replanted teeth and that HO-1 expression may be associated with PDL healing following tooth replantation in this rat model.

### Establishment of a model of injured PDLCs

To further investigate the mechanisms underlying the effects of physiological occlusal force on PDL healing *in vitro*, we developed an injured PDLCs (iPDLCs) model using direct desiccation stress to simulate cellular damage in PDL tissues of replanted teeth.

**Fig. 6:**
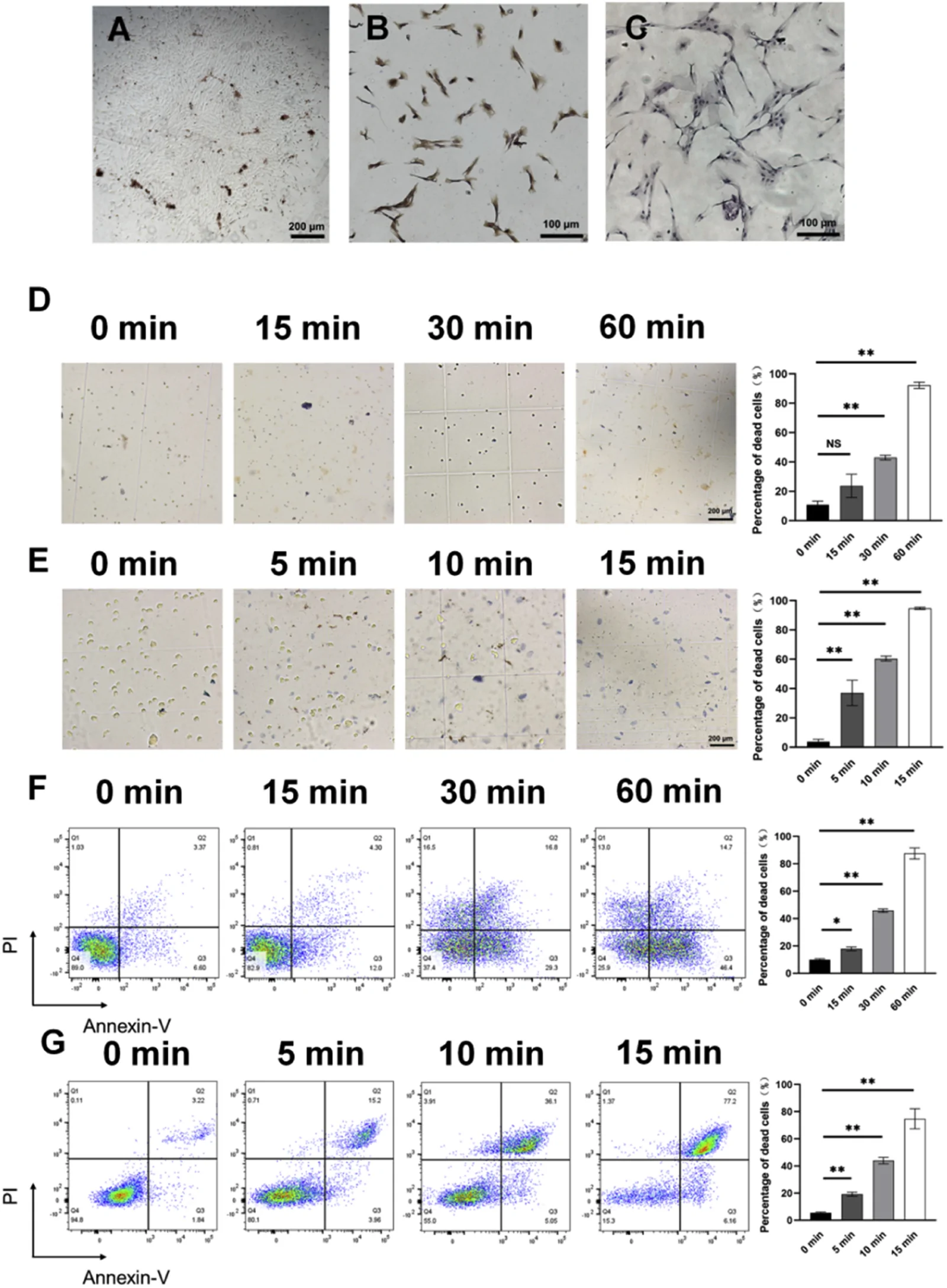
The proportion of damaged periodontal ligament cells (PDLCs) undergoing cell death increases with prolonged dry time. **A** PDLCs migrating from tissue explants (40×). **B** Immunocytochemical staining positive for vimentin expression (100×). **C** Immunocytochemical staining negative for cytokeratin expression (100×). Scale bars: a = 200 μm; b, c = 100 μm. **D** Trypan blue staining of teeth dried in air for varying durations shows an increase in cell death with longer dry times. **E** Trypan blue staining of PDLCs subjected to dry stimulation for different durations reveals an increase in the proportion of damaged PDLCs undergoing death over time. **F** Flow cytometry of teeth dried in air for varying durations demonstrates an increase in apoptosis with prolonged dry times. **G** Flow cytometry of PDLCs subjected to dry stimulation for different durations shows a higher proportion of damaged PDLCs undergoing apoptosis over time (*n* = 3). Scale bars: d, e = 100 μm; NS: *P* > .05, *P < .05, **P < .01*.* Error bars represent the mean ± standard deviation.

First, the primary cells were isolated. On day 6 of the tissue explant culture, the PDLCs migrated radially from the explant periphery, exhibiting adherent growth with a small, spindle-shaped morphology (Figure 6A). Third-passage PDLCs were subjected to immunohistochemical staining. Microscopic examination revealed that PDLCs stained positive for vimentin (Figure 6B) and negative for cytokeratin (Figure 6C). These findings, combined with the cell isolation procedure, confirmed the successful establishment of PDLCs cultures.

To establish the appropriate duration of direct desiccation stress for PDLCs, we used trypan blue staining and apoptosis detection assays to quantify cell death and apoptosis rates in PDLCs following desiccation stress, and compared them with PDLCs from the root surfaces of avulsed teeth after 30 min of dry storage. The results demonstrated that PDLCs subjected to 10 min of *in vitro* desiccation exhibited cell death (Figure 6D, E) and apoptosis (Figure 6F, G) rates equivalent to those of PDLCs from teeth dried for 30 min *ex vivo*. Therefore, 10 min of desiccation was selected as the standardized iPDLCs induction protocol to simulate the 30-minute dry storage condition of avulsed teeth.

### Cyclic tensile force upregulates HO-1 expression in iPDLCs

**Fig. 7:**
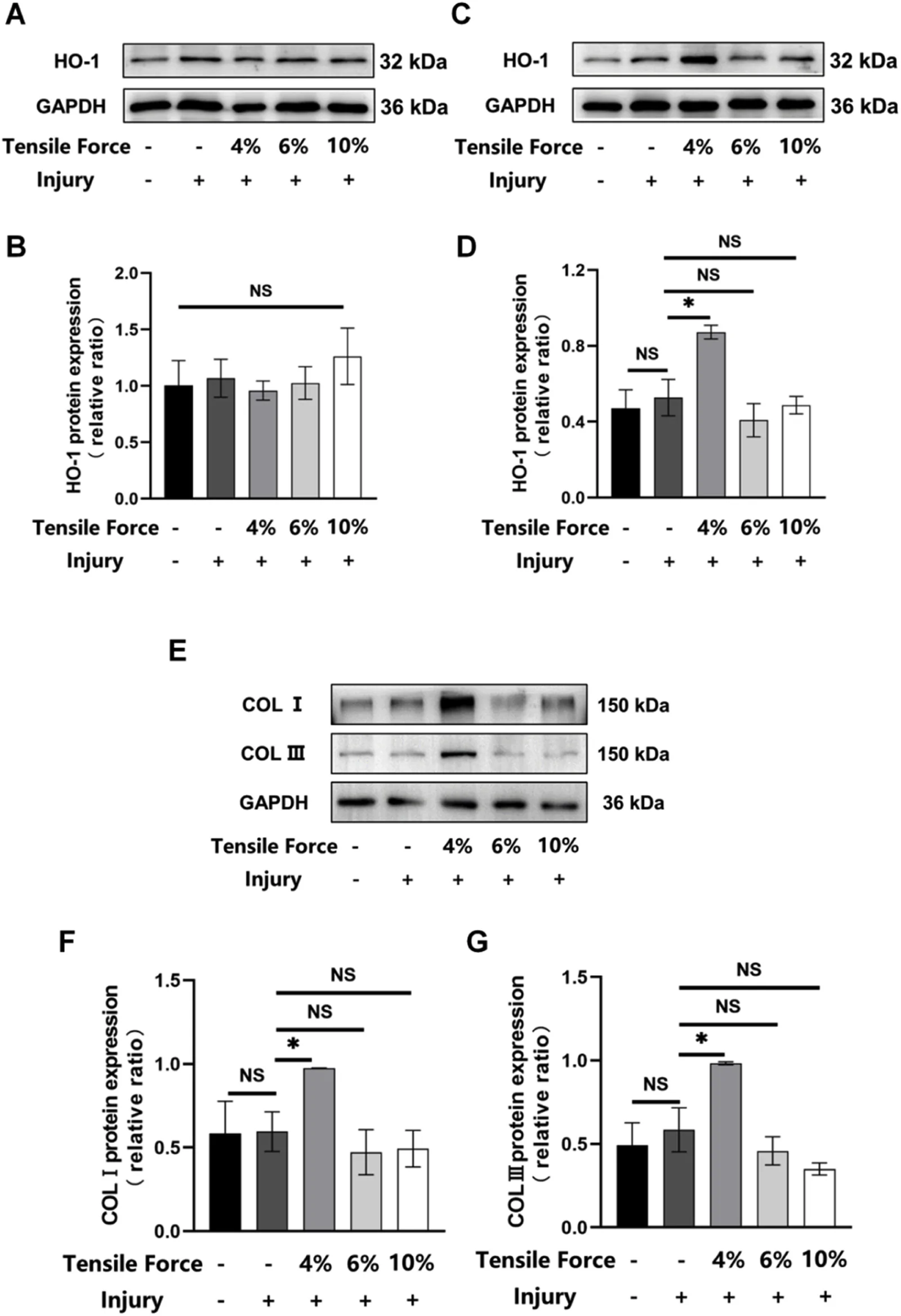
Cyclic tensile strain promotes HO-1, COL I, and COL III expression in iPDLCs. **A, B** Western blot results after 2 h of cyclic tensile strain application show no significant increase in HO-1 expression at any strain magnitude. **C, D** Western blot results after 4 h of cyclic tensile strain application reveal a significant increase in HO-1 expression at 4% strain magnitude. **E** Western blot results after 4 h of cyclic tensile strain application demonstrate altered COL I and COL III expression in iPDLCs. **F, G** Semi-quantitative analysis of western blot results after 4 h of cyclic tensile strain application. NS: *P* > .05, *P < .05. Error bars represent the mean ± standard deviation. Three independent biological replicates (*n* = 3) were performed.

To investigate the relationship between mechanical stimulation and HO-1 expression, iPDLCs were subjected to cyclic tensile force, followed by western blot analysis. After 2 h of loading, varying strain magnitudes did not induce significant differences in HO-1 expression in the three study groups (Figure 7A, B). After 4 h, a cyclic tensile force of 4% significantly enhanced HO-1 expression in iPDLCs, whereas strain magnitudes of 6% and 10% caused no differences between treated and untreated iPDLCs (Figure 7C, D). These results confirm that cyclic tensile force modulates HO-1 expression in iPDLCs in a duration- and magnitude-dependent manner, with 4-hour loading at 4% strain producing the most pronounced effect. Therefore, this magnitude and timing were selected for subsequent experiments.

### Cyclic tensile force promotes COL I and COL III expression in iPDLCs

To investigate the relationship between mechanical stimulation and collagen synthesis, we examined the expression of COL I and COL III in iPDLCs after application of cyclic tensile force. Western blotting results demonstrated that 4 h of cyclic tensile force at 4% strain magnitude significantly enhanced the expression of both COL I and COL III in iPDLCs. In contrast, strain magnitudes of 6% and 10% showed no significant differences in COL I or COL III expression compared to the control group (Figure 7E-G). These findings indicate that cyclic tensile force promotes COL I and COL III expression in iPDLCs, with a 4% strain magnitude representing optimal mechanical conditions for stimulating collagen expression. Considering the previously demonstrated regulatory effect of cyclic tensile force on HO-1 expression in iPDLCs, we ultimately selected a 4% strain magnitude with a 4-hour loading duration as the standardized mechanical parameter for subsequent mechanistic investigations.

### TGF-β1-signaling pathway mediates HO-1-induced COL I and COL III synthesis in iPDLCs

**Fig. 8:**
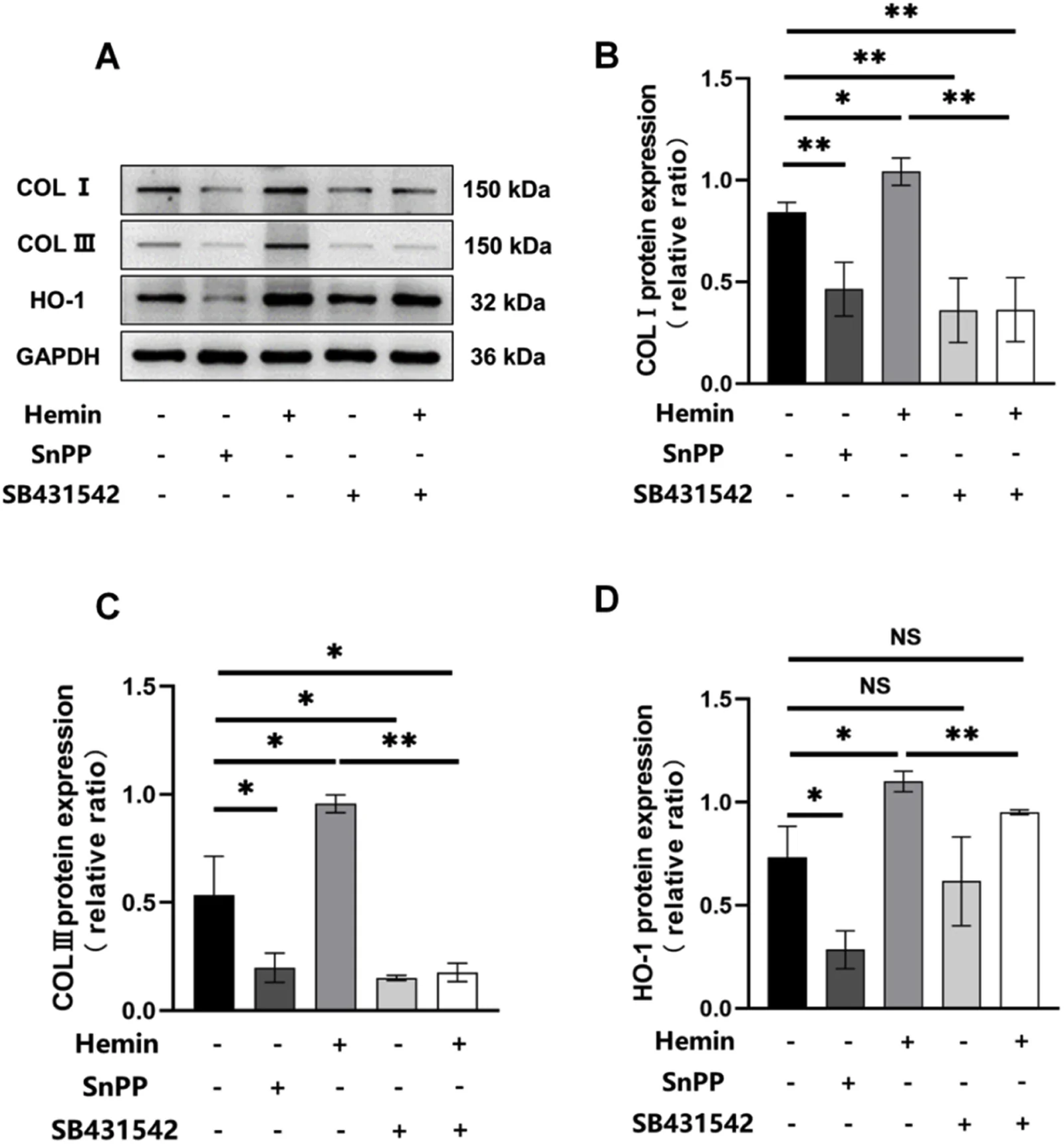
HO-1 promote COL I and COL III synthesis via TGF-β1 signaling pathway in iPDLCs. **A** Schematic of HO-1 enhance COL I and COL III production. **B-D** Western blot results show that Hemin increases HO-1, COL I, and COL III expression levels, whereas SnPP inhibits HO-1, COL I, and COL III expression in iPDLCs. SB431542 suppresses COL I and COL III expression in iPDLCs, and simultaneous treatment with Hemin and SB431542 maintains this suppression. NS: *P* > .05, *P < .05, **P < .01*.* Error bars represent the mean ± standard deviation. Three independent biological replicates (*n* = 3) were performed.

To investigate the mechanism by which cyclic tensile force regulates collagen synthesis in iPDLCs, we treated iPDLCs with HO-1 activators, HO-1 inhibitors and TGF-β1 receptor kinase inhibitor and examined the expression of related proteins. Western blot results showed that treatment with the HO-1 activator Hemin increased HO-1 protein expression in iPDLCs while upregulating the expression of COL I and COL III. In contrast, the HO-1 inhibitor, SnPP, suppressed HO-1 protein expression and reduced COL I and COL III levels. Administration of the TGF-β1 receptor kinase inhibitor SB431542 inhibited COL I and COL III expression in iPDLCs. Notably, even after co-treatment with the HO-1 activators Hemin and SB431542, COL I and COL III expression remained significantly downregulated (Figure 8). These results demonstrate that cyclic tensile force promotes COL I and COL III synthesis in iPDLCs in association with the TGF-β1 signaling pathway through enhanced HO-1 expression.

## Discussion

Occlusal force plays a crucial role in maintaining the normal morphology of PDL tissues. When occlusal force is absent, PDL tissues undergo atrophy, which is characterized by decreased numbers of PDLC and disorganized arrangement of collagen fibers.^4^^,^^28^ Masticatory forces promote PDLCs proliferation and the fibroblastic differentiation of PDL stem cells, and significantly influences collagen synthesis and distribution.^29^, ^30^, ^31^

Histologically, the healing patterns of replanted teeth can be classified into four types with PDL healing representing the most desirable outcome. The PDLCs on the root surface of replanted teeth are not only involved in the formation of the PDL but also contribute to the development of cellular cementum.^32^ Moreover, cementum is critical for resisting root resorption.^33^ Clinical studies and the latest international guidelines have confirmed that mechanical stimulation during the recovery period plays a vital role in preventing replacement resorption and promoting PDL healing in replanted teeth,but the underlying biological mechanisms require further investigation.^3^^,^^26^

The PDL, a specialized connective tissue situated between tooth roots and the alveolar bone, functions as a mechanosensory apparatus that responds to occlusal stimuli.^34^^,^^35^ In avulsion-injured periodontal tissues, occlusal deprivation disrupts the equilibrium between root regeneration and surface resorption, precipitating extensive replacement resorption that ultimately diminishes root length.^26^ Additional research indicates that occlusal forces enhance periodontal cell proliferative activity, thereby promoting healing and preventing ankylosis.^36^ In pre-replantation orthodontic treatment, where therapeutic forces augment periodontal cell proliferation capacity to improve healing outcomes and reduce resorption.^37^ Our experimental data substantiate these findings and demonstrate that the physiological occlusal force generated by mastication in rats significantly reduces replacement root resorption, preserves root length, and facilitates periodontal repair in replanted teeth. Moreover, inhibition of autophagic flux effectively reduces osteoclast differentiation and bone resorption,suggesting that occlusal force may participate in suppressing replacement root resorption by modulating autophagy-related pathways.^38^

The PDL is composed of abundant ECM, with a healthy and intact ECM serving as the foundation for its biological functions. As the predominant component of PDL, COL I not only contributes to tooth support but also participates in various physiological processes, including immune responses, wound healing, and tissue regeneration.^39^ Consequently, an occlusal force-induced increase in the proportion of COL I facilitates PDL remodeling. COL III, primarily localized in Sharpey fibers, constitutes a minor component of the normal PDL but becomes prominent in immature and healing tissues, where it is eventually replaced by COL I. This collagen subtype is associated with rapid PDL adaptation and shows marked accumulation at sites of stress concentration and active remodeling.^40^, ^41^, ^42^, ^43^ In this study, the significant increase in the proportion of COL III in the periodontal tissue at 7 days after replantation may be associated with active remodeling during the early stage of periodontal ligament healing. In contrast, the increased proportion of COL I at 28 days after replantation may be related to the transition of collagen composition following the formation of a mature PDL. Mechanostimulation studies on stretched skin tissues have demonstrated that appropriate mechanical forces significantly enhance both COL I and COL III expression to promote quality tissue regeneration, further supporting the close relationship between occlusal force-mediated PDL healing and collagen synthesis.^44^ Notably, orthodontic force studies in animal models revealed differential collagen remodeling patterns and tension zones showed increased COL I and COL III expression with new bone formation, whereas pressure zones exhibited elevated COL III but reduced COL I expression.^45^^,^^46^ Therefore, the expression of different collagen subtype in the periodontal tissue may not only be a consequence of collagen remodeling and maturation during periodontal ligament healing but also an adaptation of the tissue to environmental demands under mechanical stimulation.

Previous studies have shown that under identical H_2_O_2_-induced oxidative stress, PDLCs pre-conditioned with cyclic tensile forces exhibited markedly improved cell viability.^47^ In our investigation, HO-1 expression in the occlusion group than in the occlusal deprivation group at 7 days after replantation. HO-1 is a well-established cytoprotective factor that mitigates cell death and maintains cellular functions through its antioxidant properties.^48^ Incorporating antioxidants into tooth storage media or applying them to root surfaces can also effectively reduce PDLCs apoptosis and subsequent root resorption.^17^^,^^49^ These findings suggest that occlusal force may contribute to periodontal repair occurring via HO-1-dependent mechanisms. To further elucidate whether mechanical stimulation is associated with the functional recovery of the injured PDL via HO-1 induction, we established an *in vitro* iPDLCs model to investigate the role of HO-1 in mechanotransduction-mediated cellular rehabilitation.

The PDL is subjected to stretching during mastication. PDLCs perceive mechanical stimuli and transduce them into biological signals to regulate cellular activities.^50^^,^^51^ In PDL stem cells, cyclic tensile forces upregulate HO-1 expression and modulate cellular functions.^52^ Emerging evidence suggests PDLCs from patients with periodontitis exhibit superior functional responses under lower-magnitude cyclic strain compared to those of their healthy counterparts.^53^ Therefore, based on the mechanical specifications of our strain apparatus and published strain parameters,^54^, ^55^, ^56^ we selected strain magnitudes of 4%, 6%, and 10% for the investigation. Notably, a strain magnitude of 4%, representing a milder mechanical stimulus in our *in vitro* occlusal force simulation, induced the most significant increase in HO-1 expression after 4 h of loading. This outcome may reflect a disrupted homeostasis in iPDLCs. Prior studies demonstrated strain magnitude-dependent cellular responses. Optimal strain promotes differentiation and proliferation, whereas excessive strain induces apoptosis and suppresses ECM synthesis.^57^^,^^58^Corresponding *in vivo* observations have revealed that excessive occlusal loading impairs post-translational modifications of collagen in periodontal tissues^59^ These differential cellular responses to strain magnitude underscore the clinical importance of controlled occlusal loading during the healing of replanted teeth. Indeed, in addition to affecting collagen synthesis in PDL cells, mechanical stimulation also influences cell differentiation. Studies have shown that cyclic tensile force promotes osteogenic differentiation of PDL stem cells through the ERK/JNK pathway.^60^ In aged PDL stem cells, it also enhances mitophagy, thereby reversing the age-related decline in osteogenic differentiation capacity.^61^ These differential responses of periodontal ligament cells to mechanical stimulation warrant further investigation and exploration.

One of the primary functions of PDLCs is the synthesis and secretion of collagen to maintain ECM homeostasis. TGF-β1 is abundantly expressed in PDL tissues and plays a pivotal role in maintaining periodontal homeostasis and facilitating PDL repair and regeneration.^22^ Its expression can be modulated by mechanical stimulation. Intermittent compressive stress upregulates intracellular TGF-β1 levels, which subsequently regulate cytoskeletal organization, cell migration, and tissue homeostasis.^24^^,^^62^ Mechanistically, TGF-β1 promotes fibroblastic differentiation of PDL stem cells by upregulating COL I, α-smooth muscle actin, and periostin expression.^63^ Periostin is particularly crucial for maintaining the structural integrity and function of the PDL and serves as a key mediator in occlusal force-dependent periodontal homeostasis. Notably, mechanical stimulation significantly enhances TGF-β1-mediated regulation of periostin in PDL fibroblasts.^64^TGF-β1 primarily mediates intracellular signaling through Smad proteins, where phosphorylation of Smad2/3 can either activate or suppress downstream gene transcription.^63^ Notably, the TGF-β1/Smad3 signaling pathway can be mechanically activated by tension while simultaneously inhibiting tension-induced osteogenic differentiation.^65^ Previous studies have established the regulatory role of TGF-β1 in the collagen synthesis by PDLCs.^64^^,^^66^ In mechanically injured fibroblasts, the modulation of TGF-β1 expression can restore collagen production and ECM remodeling capacity, a process associated with the activation of the Nrf2 antioxidant system.^57^ Our findings corroborate these observations, demonstrating an integral relationship between TGF-β1 signaling pathway-mediated recovery and altered collagen synthesis capacity in iPDLCs. HO-1 is a classic downstream transcription factor of Nrf2. Studies on skin and ocular scleral tissue demonstrated HO-1 has inhibited collagen degradation by alleviating oxidative stress.^67^^,^^68^

Although this study found that physiological occlusal force upregulates HO-1 expression and promotes the synthesis of COL I and COL III in injured PDL tissue, the transitional process between COL I and COL III and their interrelationship remain to be elucidated. Moreover, the role of HO-1 expression in regulating collagen synthesis and periodontal ligament healing remains to be definitively demonstrated. In terms of animal experiments, the method of establishing an occlusal deprivation model by extracting the antagonist teeth combined with a soft diet only eliminates the occlusal force on the replanted teeth at a single level and does not allow precise quantification of the actual occlusal load borne by the replanted teeth. In experiments *in vitro*, the iPDLCs model which uses only dry storage as a injured condition, fails to fully simulate the multifactorial environment affecting the prognosis of replanted teeth. Furthermore, when cyclic tensile force is used to simulate the physiological occlusal force in rats, the quantitative conversion relationship between the *in vitro* loading conditions and the actual *in vivo* mechanical environment remains imprecise. Finally, whether HO-1 regulates other cellular functions beyond collagen synthesis in iPDLCs, as well as its interaction with TGF-β1 and the specific signal transduction mechanisms involved, requires further investigation.

## Conclusions

Physiological occlusal force is associated with periodontal ligament healing and elevated HO-1 expression in replanted teeth. Furthermore, *in vitro* experiments revealed that elevating HO-1 level can restore the collagen synthesis capacity of iPDLCs and this effect requires the TGF-β1 signaling pathway.

## Data availability

The data supporting the findings are available from the corresponding author upon reasonable request.

## Author contributions

*Experimental design*: Weiqi Jia. *Data acquisition*: Weiqi Jia, Xiaohan Liu, Chang Wu, Chuanyezi Wu. *Interpretation*: Weiqi Jia, Liming Jiang. *Drafted*: Weiqi Jia. *Critically revised the manuscript*: Weiqi Jia, Liming Jiang. *Processing*: Xiaohan Liu, Chang Wu, Chuanyezi Wu. *Analysis*: Xiaohan Liu, Chang Wu, Chuanyezi Wu. *Conceptualization and design*: Liming Jiang. *Data analysis*: Liming Jiang.

## Conflict of interest

None disclosed.
